# miR-221-5p enhances cell proliferation and metastasis through post-transcriptional regulation of SOCS1 in human prostate cancer

**DOI:** 10.1186/s12894-018-0325-8

**Published:** 2018-03-05

**Authors:** Ning Shao, Gui Ma, Jinying Zhang, Wei Zhu

**Affiliations:** 1 Department of Urology, Fudan University Shanghai Cancer Center, Shanghai, China; 2 Department of Urology, Second People’s Hospital of Wuxi, Nanjing Medical University, Wuxi, China; 3 Department of Oncology, First Affiliated Hospital of Nanjing Medical University, Nanjing, 210023 China

**Keywords:** Prostate cancer, miR-221-5p, SOCS1, Cell proliferation, Cell migration, Tumor xenograft

## Abstract

**Background:**

To investigate the effect of miR-221-5p on cell proliferaton and metastasis of human prostate cancer in vitro and vivo.

**Methods:**

We established PC3 cell lines with stable overexpression or silencing of miRNA-221-5p via lentivirus infection. miRNA-221-5p and its target gene SOCS1 expression levels in the stable cells were analyzed by real-time polymerase chain reaction (RT-PCR) and western blotting. Using luciferase reporter assays to study the relationship between miR-221-5p and SOCS1. Cell proliferative activity was measured using the MTT assay and colony formation assay. Migration ability was assessed using wound-healing assay and transwell assay. To further study the function of miR-221-5p in human prostate cancer we established nude mice xenograft model in vivo.

**Results:**

miR-221-5p regulates the proliferation, migration of prostate cancer cells in vitro and tumorigenesis in vivo by regulating socs1 expression through targeted its 3’UTR, and miR-221-5p regulates MAPK/ERK signaling pathway and EMT features in prostate cancer cells.

**Conclusions:**

Up-regulation and silencing of miR-221-5p expression in prostate cancer cells are correlated with cell proliferation, migration and tumorigenesis, which suggest that miR-221-5p plays an important role in prostate cancer progression.

## Background

Prostate cancer (PCa) is the most common malignant tumor in the human urinary system, and it is also the second major cause of death in the world. [1, 2]. Therapeutic approach includes radical prostatectomy, radiation therapy and androgen deprivation therapy (ADT) or combination therapy including ADT with radiation therapy. Many studies have confirmed that androgens play a crucial role in prostate cancer development and progression [3, 4]. ADT is an effective therapy to control prostate cancer progression by eliminating the level of androgens in the patients. However, most of patients eventually occur resistance after ADT and turn into castration-resistant prostate cancer (CRPC) [5]. Unfortunately, the majority of the patients enhance metastatic potential and the mortality of PCa patients has greatly increased [5–7]. Curable treatment method for CRPC is not established and it is not known that the mechanism of CRPC progression in detail. Additionally, many functional genes, such as tumor suppressors, oncogenes and transcription factors, have been demonstrated to play important roles in the progression of PCa [8–11]. Considering these circumstances, discovery of new therapy approach that inhibites the development of prostate cancer progression and prolongs survival time of the patients is very important in the field.

MicroRNAs (miRNAs), small non-coding single-stranded RNAs, are negative regulators for coding genes at the post-transcriptional level to be master regulators of many important biological processes, such as cell growth, invasion, metastasis, and apoptosis, etc. all [12–14]. miRNAs can bind to complementary base-pairing sequences in the 3’untranslated regions (3’UTR) of their target gene mRNA, and results in mRNA translational inhibition or degradation [15]. Lots of studies indicate that miRNAs may play important roles in a wide range of important biological processes [16].

Accumulating evidence suggests that miRNAs can function as novel tumor oncogenes or suppressors, and the deregulation of specific miRNAs involved in many important biological processes, including proliferation, invasion, apoptosis, differentiation, angiogenesis and immune response, and lead to aberrant gene expression in various diseases [17, 18]. Gene microarray data have shown the abnormal expression and paradoxical roles of miR-221-5p in human prostate cancer tissues [19–21]. In earlier research, we find that the expression of miR-221-5p is significantly different between tumor tissues and adjacent tissues of prostate cancer patients. But the molecular mechanisms of miR-221-5p and the related target genes are largely unknown. In this study, we investigated the potential functions of miR-221-5p in prostate cancer and found that miR-221-5p can specific target SOCS1 (Suppressers of cytokine signaling (SOCS) family protein, which is tumor suppressor genes [22–25]. And we investigated that miR-221-5p accelerates cell growth, migration and tumor development of human prostate cancer cells in vitro and vivo, and miR-221-5p regulates MAPK/ERK signaling pathway and EMT features in prostate cancer cells.

## Methods

### Patient samples

For verification of miR-221-5p expression by polymerase chain reaction (PCR),20 tumor tissue and adjacent tissue samples were collected from patients with prostate cancer at Second People’s Hospital of Wuxi Affiliated to Nanjing Medical University. At the time of sample collection, the histopathological types of the patient tumors were evaluated based on the pathological stages defined by the WHO. The collection of patient tumor tissues was approved by the hospital medical ethics committee, and informed consent was obtained from all patients.

### Sample collection

The tumor tissues and adjacent tissues samples were collected from prostate cancer patients. The tissues are quickly stored in liquid nitrogen and record the patient’s detailed information.

### Cell culture

HEK293T cell and Human prostate cancer cell lines PC3,DU145 were purchased from the Institute of Cytobiology, Chinese Academy of Sciences. HEK293T cells were cultured in Dulbecco’s Modified Eagle’s Medium(DMEM) and PC3 cells were maintained in F12 K medium with 10% fetal bovine serum (Thermo Fisher Scientific) at 37°Cin a humidified air atmosphere containing 5% CO_2_.

### RNA extraction and quantitative real-time

Total RNA was purified using TRIZOL reagent (Invitrogen) and reverse transcribed to cDNA according to the PrimeScript RT reagent Kit (TaKaRa). The quantification of target gene transcripts was detected by RT-PCR using SYBR Premix Ex Taq (TaKaRa) and ABI Prism 7900 sequence detection system. GAPDH was used as a reference gene to analyze the target gene quantitatively. TaqMan miRNA Kit (Applied Biosystems) were used to detect the expression level of mature miR-221-5p with U6 small nuclear RNA as an internal control. The fold change was calculated by 2^-ΔΔCt^ .

### Construction of plasmids

The 3’UTR of SOCS1 was amplified from PC3 cells cDNA and inserted into the pMIR-REPORT Luciferase vector (Ambion). And the corresponding mutant plasmid was constructed through mutations in the seed regions of the miR-221-5p-binding sites. The miR-221-5p and miR-221-5p silencing sequence (TuD RNA, Tough Decoy (TuD) miRNA inhibitor) [26] were constructed into lentivirus plasmid pLKD-CMV-G&PR-U6-shRNA, establishing stable expression cell lines.

### Lentivirus packaging and infection

miR-221-5p overexpression or silencing vector was co-transfected with the packaging plasmids pMD2.G and pSPAX2 into HEK293T cells using Lipofectamine 2000 (Invitrogen). For establishing cell lines with stable overexpression, PC3 and DU145 cells were cultured into 6-well plates, and then infected by lentivirus solution with polybrene (Sigma-Aldrich). After incubation for 72 h, the infection efficiency of lentivirus was evaluated by RT-PCR or fluorescence.

### Cell proliferation assays

Cell viability was measured using a CellTiter Aqueous assay with MTT (Sigma–Aldrich) which convert MTT into a formazan-colored product and the absorbance was measured at a wavelength of 490 nm [27]. The cell cloning ability was measured using colony formation assay. Five hundred cells were seed into 6-well culture plates and cultured at 37 °C for 7–9 d. When colony formation was visible to the naked eye, the incubation was terminated. Then the cells were stained with Crystal Violet and colonies were counted.

### Cell migration assays

For wound healing assay, cells (2 × 10^5^ cells/well) were seeded into a 6-well plates and incubated overnight. When the cells have grown to 90%, a wound was created by a micropipette tip. Then cells were cultured with serum-free medium after rinsing with PBS to remove floating cells. The wound mark were recorded at 0 h and 24 h later under a microscope (Olympus). For transwell migration assays, cells were seeded into the top transwell chambers with serum-free medium. Then the cells on the top chambers were fixed after 48 h, and cells that did not migrate were cleared by a cotton swab. The migration cells were stained by crystal violet and counted.

### Luciferase reporter assays

Bioinformatic analysis of miR-221-5p target sites was performed using TargetScan website (http://www.targetscan.org/). For experiments, HEK293T cells plated on 96-well plates were co-transfected with miR-221-5p mimics or mimics NC, and with pMIR-REPORT-SOCS1–3’UTR(WT) or mutation plasmid pMIR-REPORT-SOCS1–3’UTR(MUT) using Lipofectamine 2000 Transfection Reagent. The firefly luciferase and ranilla luciferase activities were quantified using Dual-Luciferase Reporter Assay system (Promega).

### Antibodies and immunoblotting

The antibodies purchased were as follows: anti-GAPDH antibody, anti-phospho ERK1/2 from Cell Signaling Technology; anti-total ERK1/2 from Abcam Biotechnology. Protein lysates of cells were resolved by 10% SDS–polyacrylamide gel electrophoresis (SDS–PAGE) and transferred onto PVDF membranes. After the membranes were blocked by 5% nonfat milk, the membrane was incubated with specific antibodies as well as the secondary antibodies labeled with horseradish peroxidase and visualized by chemiluminescence [28].

### Nude mice xenograft models

All animal procedures were performed in accordance to the protocols approved by the Institutional Animal Care and Use Committee at Second People’s Hospital of Wuxi Affiliated to Nanjing Medical University. All animals were obtained from Shanghai SLAC Laboratory Animal Co.,Ltd. For xenograft models, the two PC3 cell lines were contributed, including miR-221-5p silencing cell and control cell lines. Two groups of cells in the logarithmic phase of growth were trypsinized and rinsed with PBS three times. Five nude mice per group (four-week-old male, total 10 mice) were injected with a clonal population of PC3 cell (5 × 10^6^ cells) in 100ul PBS in the upper right shoulders subcutaneous. Tumor volumes were measured every other days by digital callipers when the implantations were starting to grow bigger. The animals were then euthanized by intravenous injection of potassium chloride under general anesthesia. Tumor volumes were calculated using the formula: V(mm^3^) = length × width^2/2^ [8, 28].

### Statistical analysis

Statistical significance was assessed using Student’s t test.

## Results

### miR-221-5p promotes cell proliferation of prostate cancer cells

**Fig. 1:**
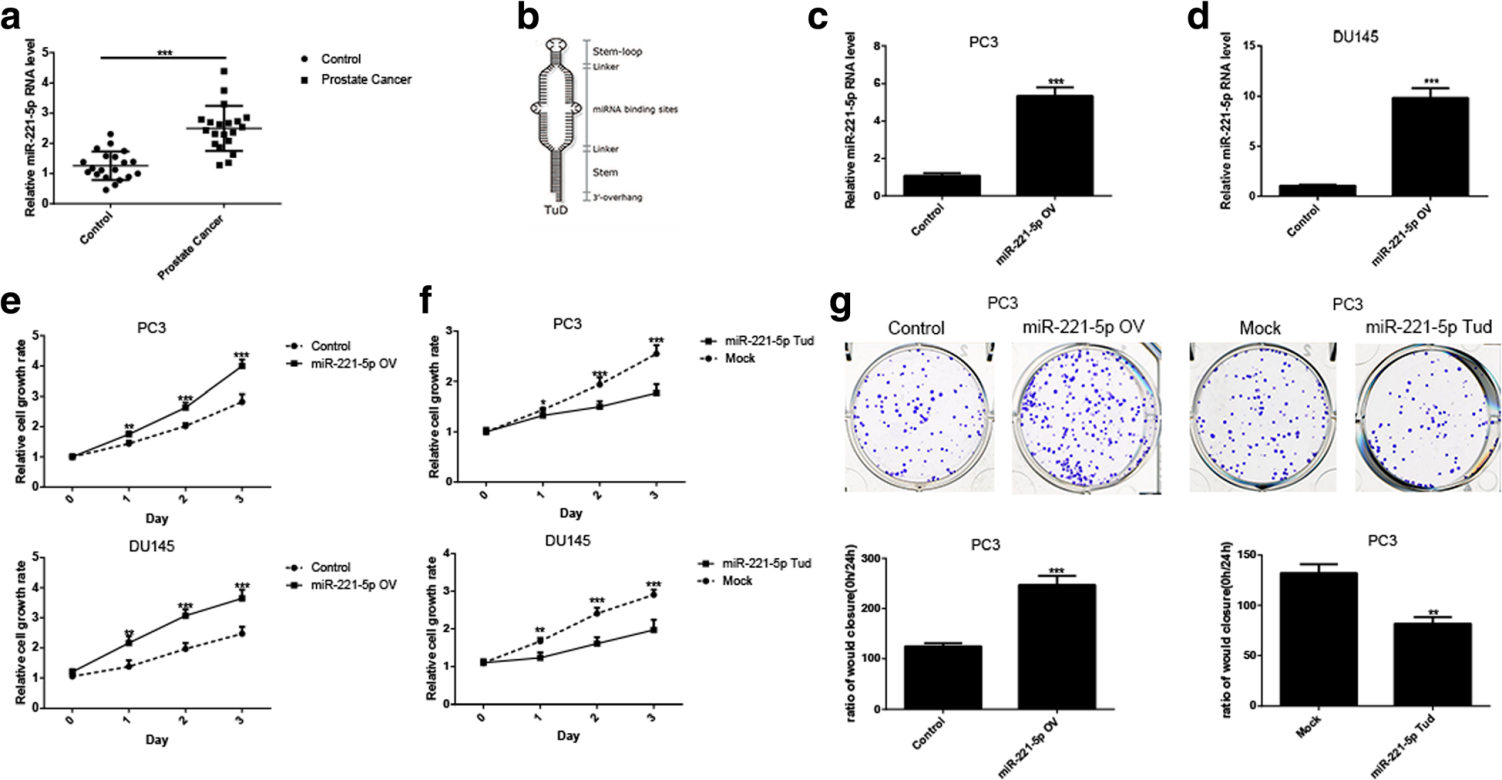
miR-221-5p regulates cell proliferation in human prostate cancer cells. **a** The expression level of miR-221-5p in tumor tissues and adjacent tissues of prostate cancer patients. **b** The mode pattern of miR-221-5p silencing system. **c**, **d** The expression level of miR-221-5p in control and miR-221-5p-overexpressing (miR-221-5p OV) prostate cancer cells as detected by quantitative RT-PCR. **e**, **f** By MTT assay, miR-221-5p overexpression or silencing regulated cell viability in PC3 cell lines (at24,48,and72 h). **g** The representative images of plate colony formation in PC3 cells.PC3 stably cells were seeded into 6-well with 500 cells per well and cultured for 7–9 days, and then colony counting. (*P < 0.05, **P < 0.01, ***P < 0.001

A previous microarray data has shown that miRNAs are differentially expressed in prostate cancer tissues, borderline tissues and that some miRNAs, including miR-221-5p, are correlated with the progression of prostate cancer. And we have found that the expression of miR-221-5p is significantly different between tumor tissues and adjacent tissues of prostate cancer patients (Fig. 1a).Nevertheless the functions of these miRNAs in the progression of prostate cancer remained unexplored. We investigated whether miR-221-5p is effective on the growth of human prostate cancer cells. We established PC3 and DU145 cell lines stably expressing miR-221-5p via lentivirus infection. Successful overexpression of exogenous miR-221-5p was confirmed by RT-PCR (Fig. 1c).We next explored the changes in cell proliferation after stably expressing miR-221-5p by MTT and colony formation assays. As shown in Fig. 1e and f, the proliferation rate of PC3 and DU145 cells was significantly increased by stably expressing miR-221-5p cells in comparison with the control cells. Congruously, colony formation ability was also significantly increased. However,using the TuD RNA (Tough Decoy RNA) (Fig. 1b), we established PC3 cell lines that the activity of miR-221-5p is closed. We found that the proliferation rate of PC3 cells was significantly reduced as well as colony formation (Fig. 1g). Collectively, these data indicated that miR-221-5p has a positive effect on the growth of human prostate cancer cells.

### miR-221-5p promotes the migration of prostate cancer cells

**Fig. 2:**
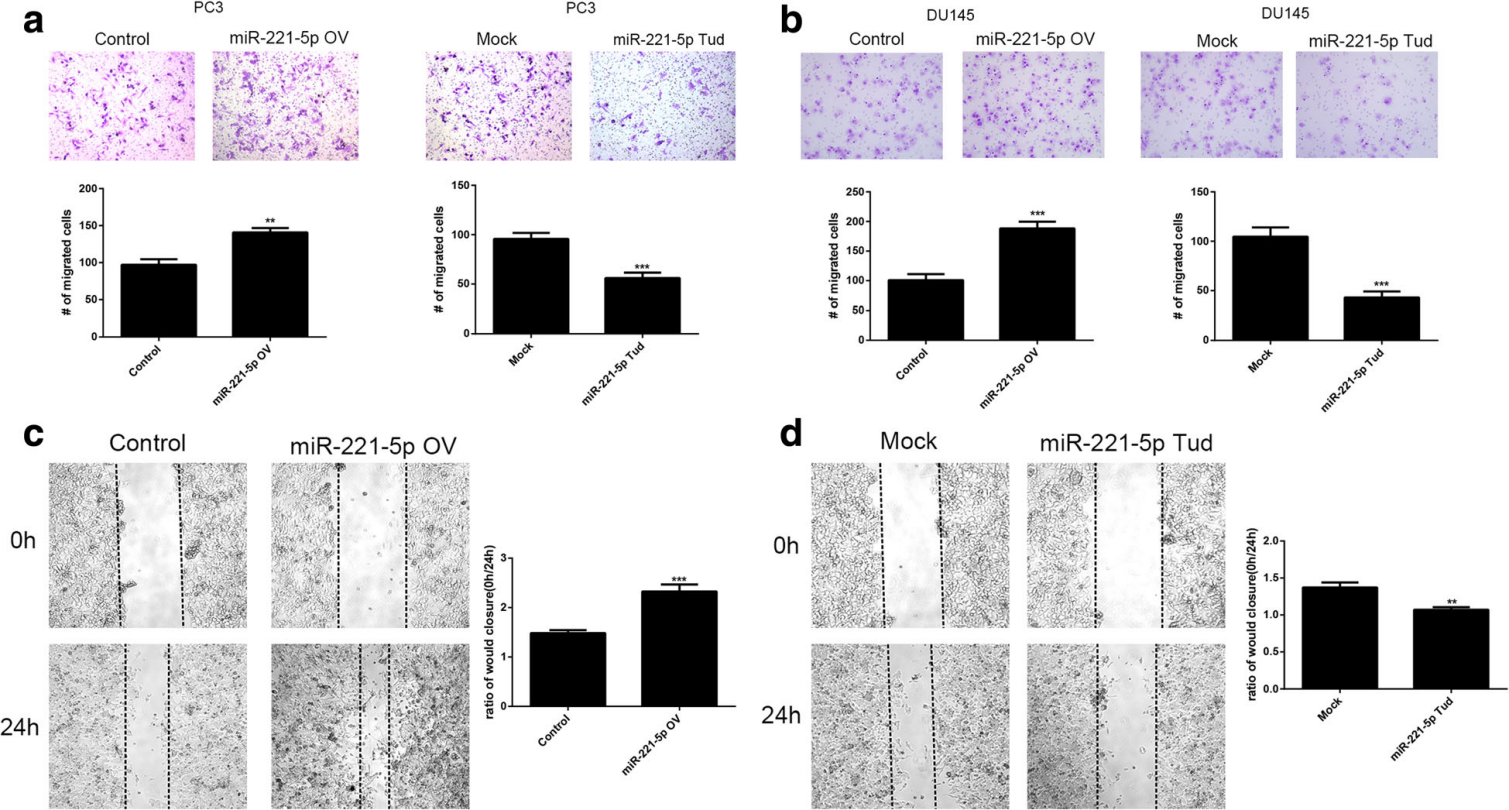
miR-221-5p regulates migration of human prostate cancer cells. **a**, **b** Effect of miR-221-5p overexpression or silencing on PC3 cells migration in wound healing assay. Representative images were was taken at 0 and 24 h after the scratch and shown in the left panel. **c**, **d** Effect of miR-221-5p on cell migration in transwell assay. The representative images of cell migration (48 h after transfection) across a membrane with 8 mm pores. The images of the staining are shown in the left panel and the number of cells are shown in the right panel. *P < 0.05, **P < 0.01, ***P < 0.001

We next investigated whether miR-221-5p regulates cell migratory ability in prostate cancer cells. The transwell assay further confirmed that miR-221-5p regulates the migration of prostate cancer cells (Fig. 2a and b). Consistently, in wound healing assay, the migration rate was significantly increased with miR-221-5p overexpression (Fig. 2c). However, the migration rate was significantly reduced in the wound healing assay and the transwell assay when we silencing the activity of miR-221-5p by TuD RNA lentivirus (Fig. 2d).In conclusion, these data confirmed that miR-221-5p promotes the migration of prostate cancer cells.

### miR-221-5p down-regulates SOCS1 expression by targeting its 3’UTR

**Fig. 3:**
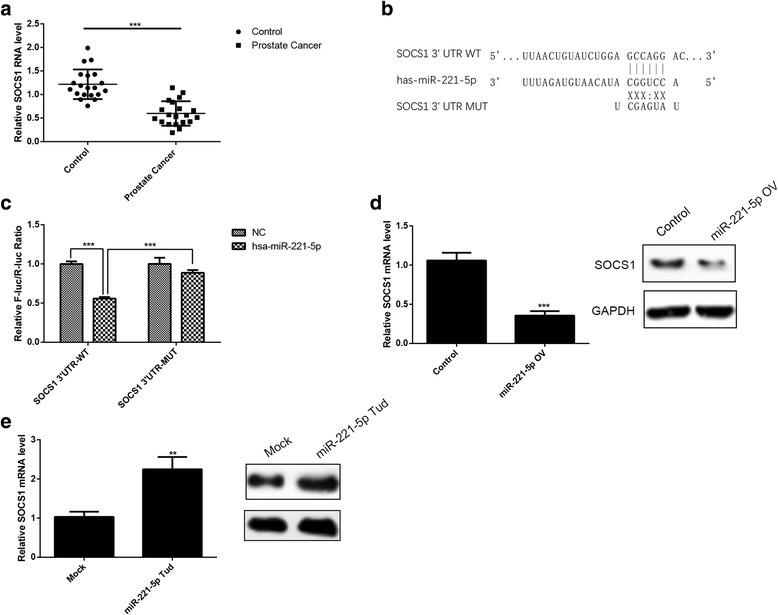
SOCS1 is a direct downstream target for miR-221-5p. **a** The expression level of SOCS1 in tumor tissues and adjacent tissues of prostate cancer patients. **b** Model of the construction of wild-type or mutant SOCS1 3’UTR vectors. **c** Luciferase activity assays of luciferase vectors with wild-type or mutant SOCS1 3’UTR were performed after co-transfection with miR-221-5p mimic or negative control (NC). The luciferase activity was normalised to Renilla luciferase activity. **d**, **e** Western blot assays of SOCS1 protein in PC3 cells after infection with miR-221-5p overexpression or silening lentivirus. *P < 0.05, **P < 0.01, ***P < 0.001

SOCS1 as a tumor suppressor gene has been reported in previous researches, and found that SOCS1 has a lower expression level in patients with prostate cancer tissues than adjacent tissues (Fig. 3a). By TargetScan and miRBase bioinformatics analyses, the 3’UTR of SOCS1 were identified as the potential binding site of miR-221-5p (Fig. 3b). To determine whether SOCS1 was regulated by miR-221-5p through direct binding to its 3’UTR, we inserted PCR products containing wild-type or mutant SOCS1 3’UTR binding sites into the pMIR-REPORT Luciferase vector. The luciferase assays showed that miR-221-5p could significant reduce the luciferase activities of the 3’UTR of SOCS1, but the luciferase activity was not significant changed when SOCS1 3’UTR binding sites are mutated (Fig. 3c). This study indicates that miR-221-5p may suppress the expression of SOCS1 by targeting the binding 3’UTR sites of the SOCS1. Next, by RT-PCR and western blotting analysis, we found that overexpression of miR-221-5p significantly suppressed SOCS1 expression but silencing of miR-221-5p increased SOCS1 expression (Fig. 3d and e).

### miR-221-5p regulates MAPK/ERK signaling pathway and EMT features in prostate cancer cells

**Fig. 4:**
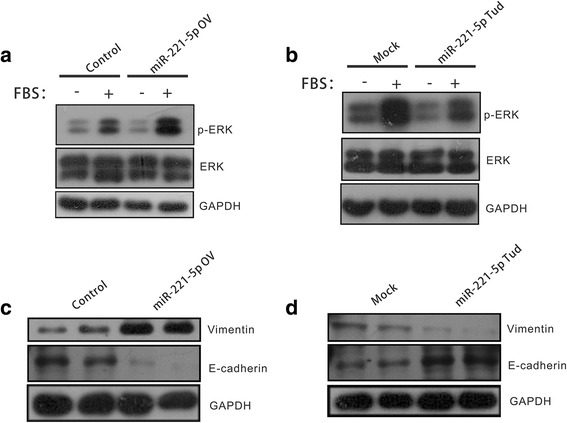
miR-221-5p regulates MAPK/ERK signaling pathways and affects EMT features in human prostate cancer cells. **a** Overexpression of miR-221-5p enhances MAPK/ERK signaling pathways. After serum starvation for 16 h, PC3 cells with overexpression of miR-221-5p or control were stimulated with 10% FBS for 20 min and the cells were harvested for immunoblotting. **b** Silencing of miR-221-5p inhibits MAPK/ERK signaling pathways. PC3 stably cells of silencing miR-221-5p or control were serum-starved for 16 h, and cells were stimulated with 10% FBS for 20 min. Western blot analysis of MAPK/ERK signaling pathways proteins in the cell samples. **c**, **d** miR-221-5p promotes EMT features and regulates the expression of mesenchymal marker vimentin and epithelial marker E-cadherin

The Ras/Raf/MEK/ERK signaling pathway plays an important role in cell proliferation. We next explored whether MAPK/ERK signaling pathway was also affected by miR-221-5p in prostate cancer cells. As expected, the serum was able to stimulate MAPK/ERK signaling pathways shown as increased phosphorylation of ERK in PC3 cells. We discovered that the phosphorylation of ERK was observably increased by overexpression of miR-221-5p in the prostate cancer cells (Fig. 4a). In contrast, silencing of miR-221-5p could inhibit serum-induced phosphorylation of ERK (Fig. 4b). These data indicated that Ras/Raf/MEK/ERK signaling pathway was regulated by miR-221-5p, likely explaining its effects on tumor-promoting activity in prostate cancer cells.

Epithelial-mesenchymal transition (EMT), a critical process for tumor migration and metastasis, was increased that shown as decrease of epithelial marker E-cadherin and increase of mesenchymal marker vimentin by overexpression of miR-221-5p (Fig. 4c). On the contrary, silencing of miR-221-5p promoted the expression of E-cadherin while suppress the expression of vimentin (Fig. 4d).

### miR-221-5p promotes prostate cancer xenograft growth in vivo

**Fig. 5:**
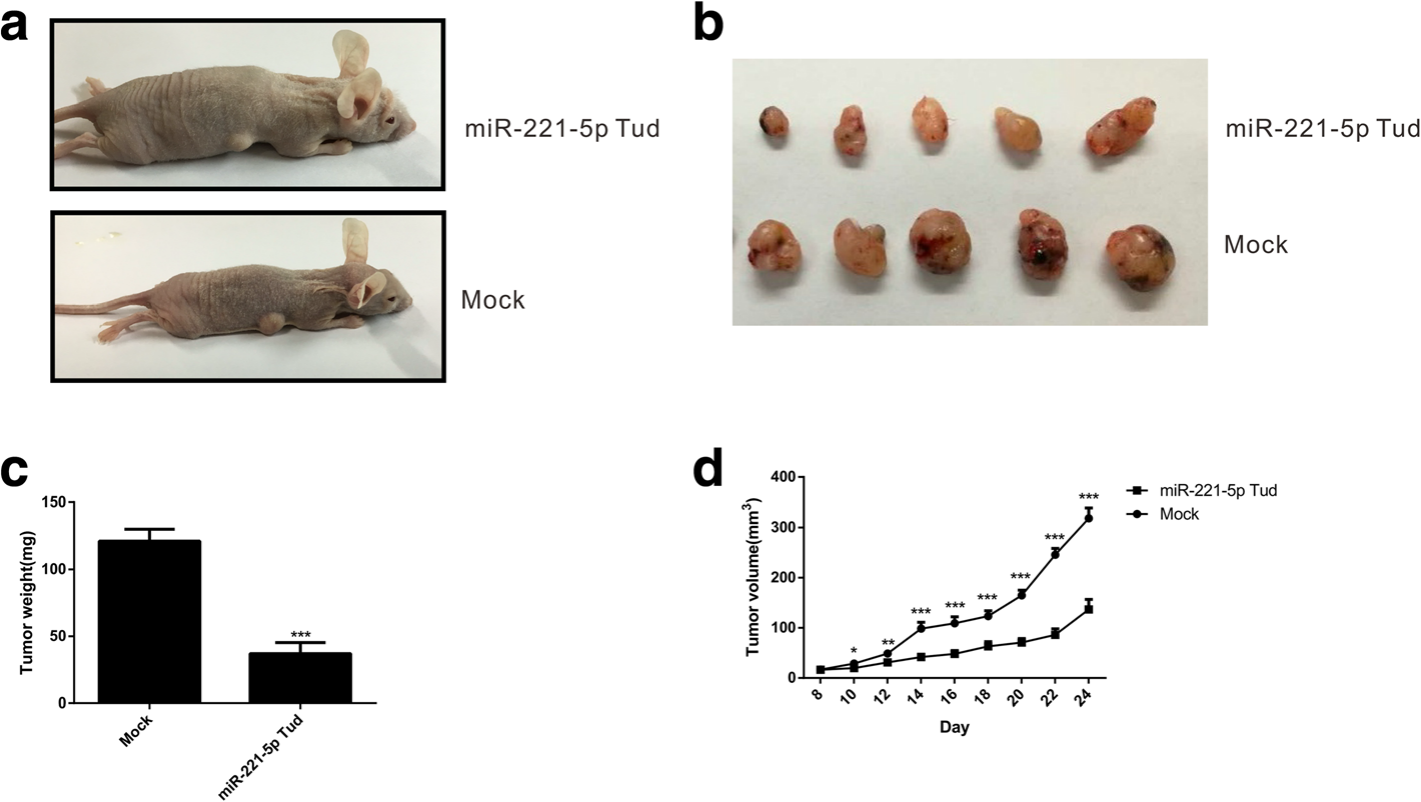
Silencing of miR-221-5p enhances the growth of PC3 cell xenografts in nude mice model. **a** The subcutaneous tumors xenografts in nude mice derived from the silencing of miR-221-5p clones were smaller in size than control clones(*n* = 5 each group). **b** Images of the tumors isolated from the nude mice(*n* = 5 each group). **c** The weight of the tumors. **d** Tumor volume as calculated according to the formula: V(mm^3^) = length × width^2/2^. *P < 0.05, **P < 0.01, ***P < 0.001

In order to further demonstrate the tumor enhance activity of miR-221-5p in prostate cancers, we established a xenograft model to investigate the effects of miR-221-5p on tumor growth. PC3 cells, silencing of miR-221-5p or its control, were implanted into the nude mice (4 weeks). Then, the mice were sacrificed in 24 days when the tumor formation have significant difference (Fig. 5s and b). The growth of the PC3 cells in the nude mice as measured by tumor volume and tumor weight was significantly increased by silencing of miR-221-5p (Fig. 5c and d). Therefore, this clearly indicated that miR-221-5p has an effective activity to promote the xenograft of prostate cancer in vivo.

## Discussion

miRNAs have been confirmed as important regulators of gene expression at the post transcriptional level, and these small non-coding RNAs molecules regulate a wide range of physiologicaland developmental processes [12–14]. Over the past several years, its mechanism become clear that regulates target gene expression of miRNAs contribute to the pathogenesis of most human cancers, where miRNAs has been proved to function as important regulators in tumorigenesis and development [13]. miRNA can regulate a variety of target genes, thus affecting the different physiological functions of cells. miRNA has obvious specificity in different cell, tissue and even different stages of tumor development, which makes miRNA play a complex and important role in the development of tumor [15–18].

Our studies have provided evidence that miR-221-5p can inhibit the expression of SOCS1 to control tumor proliferation, migration and tumorigenicity of prostate cancer cells both in vitro and in vivo. But Coarfa et al. [21] examined some publicly available, independent sets data and found that many of SIM-miRNAs were significantly downregulated in primary PC (compared with normal prostate),including miR-221-5p. Coarfa et al. found that miR-221-5p was associated with worse BCR-free survival in Taylor et al. [29] (GSE21036) according to their individual miRNA z-score (compared with normal prostate tissue).The results of our study with Coarfa et al. are inconsistent, and in order to find out why the results were inconsistent, We measured our collection of prostate cancer clinical sample, and found that in some tumor samples, the expression of miR-221-5p in the adjacent tissues was elevated and decreased in the cancer tissue, which is consistent with Coarfa et al’s findings and indicated that the patient had obvious heterogeneity, but in the whole clinical data, miR-221-5p was positively correlated with prostate cancer. This suggests that miR-221-5p may have different effects at different stages of prostate cancer, but the number of prostate cancer samples currently collected is only 20, which is not enough to illustrate the problem. We will also collect more patient samples in subsequent trials to analyze the relationship between mir-221-5p and prostate cancer during different stages of development.

In this study, we found that miR-221-5p can specific target SOCS1, which is tumor suppressor genes,and suppress SOCS1 protein expression in PC3 and DU145 cells. But studies of Coarfa et al. show that miR-221-5p suppressed AR protein expression in LNCAP cells. This phenomenon further illustrates the complexity and specificity of miRNA functions in different tissues and cells.In subsequent studies, we will focus on the differences of miRNA in prostate cancer patients sample at different stages of tumor development or the different therapies, to discover more valuable information in the diagnosis and treatment of prostate cancer.

At the cellular level, by establishing the stably expression cell lines of overexpression or silencing miR-221-5p, we found that miR-221-5p regulates the cell proliferation, colony formation and migration of human prostate cancer cells. At the animal level, silencing of miR-221-5p inhibited significantly the tumorigenesis of prostate cancers in nude mice.

Given that the expression of SOCS1 is regulated at post-transcriptional level by miR-221-5p, detection the expression of miR-221-5p in cancer tissues would discover an effective approach to evaluate miR-221-5p as a potential prostate cancer biomarker. Extracellular signal regulated kinase (ERK) is a kinase regulating cell survival, growth and proliferation by promoting proline-induced protein phosphorylation, a critical process that controlling cell proliferation and metastasis [14]. SOCS1, a key inhibitory molecule of MAPK/ ERK signaling, can inhibit cell proliferation by suppressing cell cycle progression, promoting cell apoptosis, or promoting tumor cell metastasis and invasion when it is expressed aberrantly in cells [22–25]. In our studies, luciferase assays, qRT-PCR and western blotting demonstrated that miR-221-5p can target SOCS1 and regulates the expression in cells level. And miR-221-5p is able to regulate Ras/Raf/MEK/ERK signaling cascades in prostate cancer cells and such enhancement likely underlies its tumor-promoting activity in prostate cancer cells.

EMT as a critical step for tumor migration and metastasis has been demonstrated that miR-221-5p regulates EMT features in prostate cancer cells. As miR-221-5p is able to promote migration of prostate cancers, the association of miR-221-5p/SOCS1 with metastasis and EMT need to be addressed in the future. Theoretically, silencing the tumor promoting activity of miR-221-5p can stand out as an effective approach to inhibit cancer progression. Discovery of chemical molecules or other ways to regulate tumor promoting activity of miR-221-5p will have more effective to control tumor cells and inhibit tumor cell proliferation, tumor migration/metastasis. Future studies are needed to explore the association with miR-221-5p and SOCS1, and whether miR-221-5p/SOCS1 pair can be used as a new biomarker to diagnosis of prostate cancer, and whether it can be as a novel therapeutic target in prostate cancer treatment.

## Conclusion

In conclusion, we find a new miR-221-5p/SOCS1 pair that may play an important role in progression of prostate cancer. And we also confirm that miR-221-5p enhances cell proliferation and metastasis through post-transcriptional regulation of SOCS1 by vitro and vivo experiments in human prostate cancer.

## Abbreviations

3’UTR: 3’untranslated regions
ADT: Androgen deprivation therapy
CRPC: Castration-resistant prostate cancer
EMT: Epithelial-mesenchymal transition
ERK: Extracellular regulated protein kinases
GAPDH: Reduced glyceraldehyde-phosphate dehydrogenase
MAPK: Mitogen-activated protein kinase
MUT: Mutation
OV: Overexpression
PCa: Prostate cancer
RT-PCR: Quantitative real-time PCR
SOCS1: Suppressers of cytokine signaling (SOCS) family protein 1
TuD RNA: Tough Decoy (TuD) miRNA inhibitor
WT: Wild type
